# Neurosurgery specialty training in the UK: What you need to know to be shortlisted for an interview

**DOI:** 10.1016/j.amsu.2020.07.047

**Published:** 2020-07-28

**Authors:** Giorgos Solomou, Suzanne Murphy, Soham Bandyopadhyay, Hugo Layard Horsfall, Midhun Mohan, Aswin Chari, Saurabh Sinha, Nigel Mendoza

**Affiliations:** a Keele University Medical School, Stoke on Trent, UK; b UCD School of Medicine and Medical Science, Ireland; c Oxford University Medical School, UK; d Division of Neurosurgery, Department of Clinical Neurosciences, Addenbrooke's Hospital and University of Cambridge, Cambridge, UK; e Academic Division of Neurosurgery, Cambridge University Hospitals NHS Foundation Trust and University of Cambridge, UK; f Department of Neurosurgery, Great Ormond Street Hospital, Institute of Child Health, University College London, UK; g Sheffield Children's NHS Foundation Trust and Sheffield Teaching Hospitals NHS Foundation Trust, UK; h West London Neurosciences Unit, Charing Cross Hospital NHS Trust, UK

**Keywords:** Neurosurgery, Surgery, Training

## Abstract

Neurosurgery is one of the most competitive specialties in the UK. In 2019, securing an ST1 post in neurosurgery corresponds to competition ration of 6.54 whereas a CST1 post 2.93. Further, at ST3 level, neurosurgery is the most competitive. In addition, the number of neurosurgical training posts are likely to be reduced in the coming years. A number of very specific shortlisting criteria, aiming to filter and select the best candidates for interview exist. In the context of the high competition ratios and the specific shortlisting criteria, developing an interest in the neurosciences early on will allow individuals more time to meet the necessary standards for neurosurgery. Here, we aim to outline the shortlisting criteria and offer advice on how to achieve maximum scores, increasing the likelihood to be shortlisted for an interview.

## Highlights

• Neurosurgery is one of the most competitive specialities in the UK.
• At ST3 level neurosurgery is the most competitive surgical speciality in the UK.
• Leadership and management skills are desirable skills in the NHS.

## 1 Introduction

Neurosurgery is one of the most competitive specialties in the United Kingdom (UK). In 2018, there were 152 applicants for 34 specialty training year one (ST1) neurosurgery places and 12 applications for just 2 ST3 posts [1,2]. In 2019, the number of ST1 places were reduced to 24, of which 157 applicants applied for. This is due to the growing number of post specialty training neurosurgeons who do not have a consultant post [3]. In fact the number of surgeons who have completed their CCT in neurosurgery but who do not have a consultant post has risen from 26 to 43 between 2015 and 2018 [3]. Comparatively, there were 1870 applications for 636 Core Surgical Training (CST) posts in 2018, and 1896 applicants for 648 places in 2019. This resulted in a competition ratio of 2.94 (2018), and 2.93 (2019) for CST year 1, compared to a ratio of 4.47 (2018) and 6.54 (2019) for neurosurgery. In 2019, at ST3 level, cardiothoracic, oral and maxillofacial, paediatric, plastic, trauma and orthopaedic, general and vascular surgery and urology had competition ratios of 5.00, 0.96, 2.56, 3.92, 3.11, 2.16 and 2.07 respectively. Neurosurgery had a competition rate of 7.00 making it the most competitive at ST3 entrance.

**Fig. 1:**
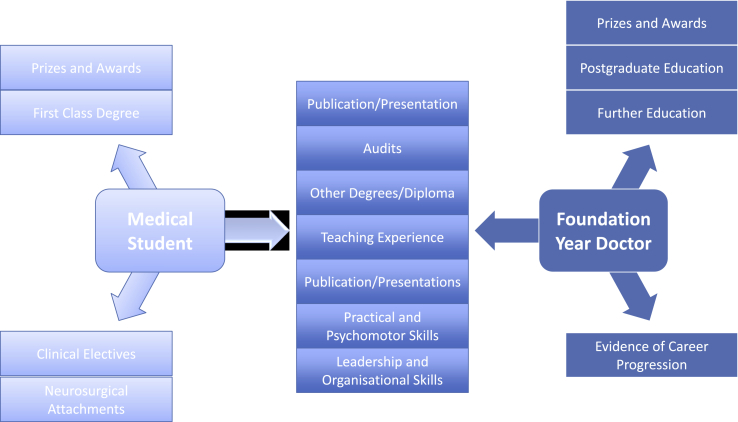
Chart showing the specific domains to work on based on the career progression. The list in the middle outlines a numbers of domains that an aspiring neurosurgeon can work on from early undergraduate years to foundation training years.

The national Neurosurgery Selection Board (NNSB) have a set of very specific shortlisting criteria, aiming to select the best candidates for interview (see Fig. 1). These criteria stipulate that published papers, presentations and audits should be tailored towards an area related to the clinical neurosciences [4], whereas the shortlisting criteria for CST1 are non specific [5]. Since over 50% of applicants are rejected prior to the interview stage, the shortlisting stage is critical for success in securing a neurosurgical post [6]. In addition, the overall shortlisting score contributes towards the final post interview selection centre score. In the context of the high competition ratios and the specific shortlisting criteria, developing an interest in the neurosciences early on will allow individuals more time to meet the necessary standards for neurosurgery.

Here, we aim to provide insight into the neurosurgical national selection application process, focusing specifically on how prospective applicants may focus their time and effort to yield the highest level of achievement for every domain of the selection criteria and thus maximise their shortlisting score.

### 1.1 Overview of shortlisting process

Selection for neurosurgical training happens through a national selection process that takes place on an annual basis within the Yorkshire and Humber Deanery [4,6]. Candidates are shortlisted for interview based on a standardised scoring system. An online application through Oriel that assesses your relevant experience accounts for 60% of the overall shortlisting score [7]. Multi-Specialty Recruitment Assessment (MSRA), a computer based exam that assesses situational awareness, professional awareness and broad clinical acumen, accounts for the remaining 40%. Candidates are offered an interview based on their shortlisting scores. The standardised scoring system covers a range of domains, with each candidate eligible to score between 0 to 2–3 points maximum in each for a maximum overall score. Each application is independently assessed and scored by two assessors.

### 1.2 Shortlisting domains

It is important to differentiate between undergraduate and postgraduate achievements and experience whilst considering the neurosurgical application. The first two domains are devoted to the undergraduate career. These include:

i) educational achievements
ii) clinical attachments/electives undertaken in neurosurgery or neurosciences whilst a medical student.

Both undergraduate performance in medical school and winning academic prizes are indicative of a hard-working, organised individual who has a high level of motivation; attributes that can dictate the success of a resident during training [8]. The more academically accomplished an individual is, the greater their chances of maximising their score in these domains. For example, winning a national award or placing in the top deciles might earn full points. Being a top decile medical student requires devotion and thus has to be consistently pursued. Aiming to apply for a national award/prize requires strategic planning months before as well as realistically recognising the probability of wining such an award. Presenting at a national conference often requires developing a high-impact research project over a long time period. A similar vein of thought is relevant for applying for student research grants and/or essay competitions. Someone needs to honestly assess their owns strengths and weakness and pursue opportunities that are more likely to yield desirable outcomes. As previous studies have suggested, exposure to neurosurgery via electives and clinical attachments not only show commitment but can also challenge common misconceptions and increase awareness about the specialty, thus increasing the likelihood the applicant has taken a well-informed decision to pursue a career in neurosurgery [[9], [10], [11]]. It is implied that those who develop an interest towards neurosurgery as a student are in a better position to maximise points devoted to undergraduate career, which may give them an edge during the shortlisting process.

Seven domains focus on previous or further educational achievements:

i) undergraduate degrees
ii) other degrees and diplomas
iii) higher degrees (PhD/MD/MS)
iv) publications
v) presentations
vi) audits
vii) postgraduate academic awards and prizes

An undergraduate Master's degree and first class Bachelor's degree will gain maximum points. One should carefully choose an extra degree to pursue, which will increase the likelihood of yielding presentations and publications as a first author versus co-author. Publications related to clinical neurosciences are looked upon favourably, especially if one is the first-named author, as that reflects major input to the manuscript. Being first-named author in more than 3 papers relevant to the neurosciences will earn maximum points.

First author manuscripts requires long term planning. Keen individuals are usually offered a variety of opportunities to get involved with. One should assess carefully, which opportunities are more likely to yield first named author publications. This can be done by assessing the academic output of potential supervisors and other group members. It is worth mentioning that taking on new projects a few months before the deadline of application might not be in favour. Being a co-author regardless of the number of papers will more likely earn less points.

Presentations when delivered orally at national/international level are preferred, likely reflecting the impact of research conducted. An applicant should be mindful of the application deadline and submit abstracts to conferences months before the application. Ideally, 3 or more oral presentations can score full points. Similarly, clinical audits and quality improvement strategies show intend to improve patient care and monitor clinical practise; an attribute highly desirable to any doctor working for the NHS. Leading the design and execution of such strategies highlight the initiative and motivation of an applicant, contrasting with those that contribute to audits that are already in place. Full cycle audits and quality improvement projects with evidence demonstrating that the work has changed practice are highly likely to score maximum points. It is important to avoid overloading oneself with multiple audits that are unlikely to be completed and re-conducted.

Additional postgraduate degrees and qualifications, for example passing the part A examination of the Membership of Royal College of Surgeons (MRCS) can score maximum points. A number of applicants sit the MRCS part A exam within the first year post-graduation. A lot of knowledge is transferrable and will more likely remain fresh the following months after final year medical school examinations. Postgraduate certificates are also commonly pursued by candidates and can also score points. A higher doctoral degree, comprised of full-time research can yield three extra points, whilst a degree in progress might qualify for fewer points.

The final four domains explore the candidate's experience in teaching, their organisational and leadership skills, engagement with postgraduate clinical courses, as well as their practical and psychomotor skills.

Teaching is a critical part of medicine, with an expectation that each generation will teach the following [12]. This is particularly true of surgical specialties. Developing/leading courses/workshops and showing continued engagement in regular teaching activities increases the likelihood of scoring maximum points.

Leadership and management skills are highly desirable in an ever-evolving NHS. Neurosurgery is a career in which a trainee will have to lead a team in the operating theatre as well as in the ward. Honing skills, such as team working, troubleshooting and escalating appropriately, communicating with people from various backgrounds are highly desirable abilities that can be demonstrated by being involved with National societies/bodies. Chairing or having similar leading roles can score maximum points.

A neurosurgical trainee not only has to be a good leader but also a highly skilled surgeon. Through playing musical instruments or sports, psychomotor skills, dexterity and physical stamina can be demonstrated. Adopting a similar approach to a musician for developing surgical skills may also improve physiological and psychological related issues [13]. Additionally, the dedication and time required to become a skilled sportsperson indicates values such as commitment, effort and dedication. All desirable traits in neurosurgical trainees. Further, having a hobby that can help destressing and contribute towards a balanced lifestyle is also seen as a positive trait.

Finally, applicants will be assessed for their engagement with postgraduate clinical courses. Early engagement exemplifies a commitment to professional development, with applicants increasing their marks by attending courses such as Basic Surgical Skills, Advance Trauma Life Support or Care of the Critically Ill Neurosurgical Patient. Applicants may also consider their own contribution to training at an undergraduate or postgraduate level, once again highlighting their abilities as teachers and leaders.

### 1.3 The interview

Shortlisted applicants attend an interview comprising of six 15 min stations, which examine the candidates’ capabilities across a wide range of skills. Problem-solving and judgement under pressure are two fundamental skills within medicine and have been cited frequently in research as essential skills for a neurosurgeon [14]. Many neurosurgical patients present through the emergency department or require acute neurosurgical intervention whilst being treated for another issue [15]. Such unpredictability means essential traits of a trainee are to be flexible and reactive, adapting to changing patient needs. Ultimately, trainees must be able to prioritise and re-prioritise dynamically. Effective communication skills and professional integrity have been widely reported to improve patient satisfaction and clinical outcomes [[16], [17], [18], [19]]. As such, they are a cornerstone of most medical school curricula and are essential to scoring well at interview. The aforementioned interview skills can be honed by applicants working to achieve excellence throughout their undergraduate and postgraduate career. Appointments are then offered to the highest scoring applicants [4].

## 2 Conclusion

An awareness and engagement with the relevant domains of national selection from an early point in an applicant's medical career will help prepare them for the application process by ensuring they can score the maximum number of points to secure their place at interview. However, developing excellent clinical and communication skills from the first day in medical school is what will get anyone through the interview stage and securing their first job as a neurosurgical trainee.

## Authorship

Georgios Solomou and Suzanne Murphy contributed equally to this paper.

## Provenance and peer review

Not commissioned, externally peer reviewed.

## Ethical Approval

N/A

## Funding

No funding was received for this research.

## Author contribution

Georgios Solomou and Suzanne Murphy contributed equally to this paper. They wrote the main body of the paper.

The other authors were equally involved with reviewing and editing the document.

All authors were involved with discussing the paper and deciding on the format.

## Consent

N/A

## Registration of research studies

Name of the registry:

Unique Identifying number or registration ID:

Hyperlink to your specific registration (must be publicly accessible and will be checked):

## Guarantor

Suzanne Murphy and Georgios Solomou

## Declaration of competing interest

Aswin Chari is supported by a Great Ormond Street Hospital (GOSH) Children's Charity Surgeon Scientist Fellowship and the 10.13039/501100000272National Institute for Health Research GOSH Biomedical Research Centre
